# Supplementary material for: DMRforPairs: identifying Differentially Methylated Regions between unique samples using array based methylation profiles
Source: BMC Bioinformatics. 2014 May 15;15:141. doi: 10.1186/1471-2105-15-141 (PMC4046028; doi:10.1186/1471-2105-15-141)
Supplement: Additional file 2 — DMRforPairs output for the comparison of A431-MCF7 and NA17018-NA17105. Please start from the HTML files in each folder. Available via the BMC Bioinformatics website. [file 1471-2105-15-141-S2.zip › 1394847754114233_MOESM2_ESM/A431_MCF7/figures/3848.pdf]

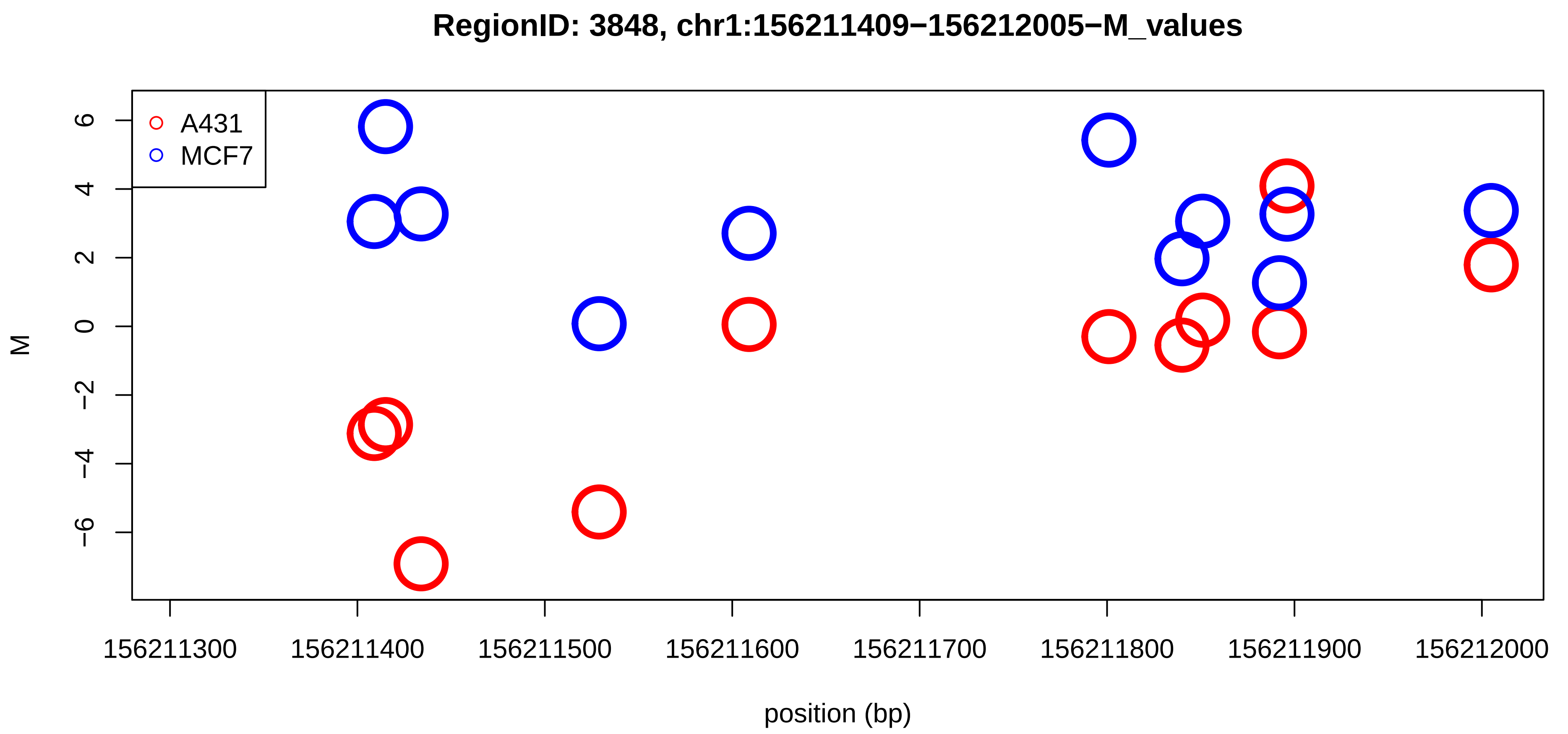
RegionID: 3848, chr1:156211409−156212005−M_values
A431
MCF7
M
6
4
2
0
−2
−4
−6
156211300
156211400
156211500
156211600
156211700
156211800
156211900
156212000
position (bp)

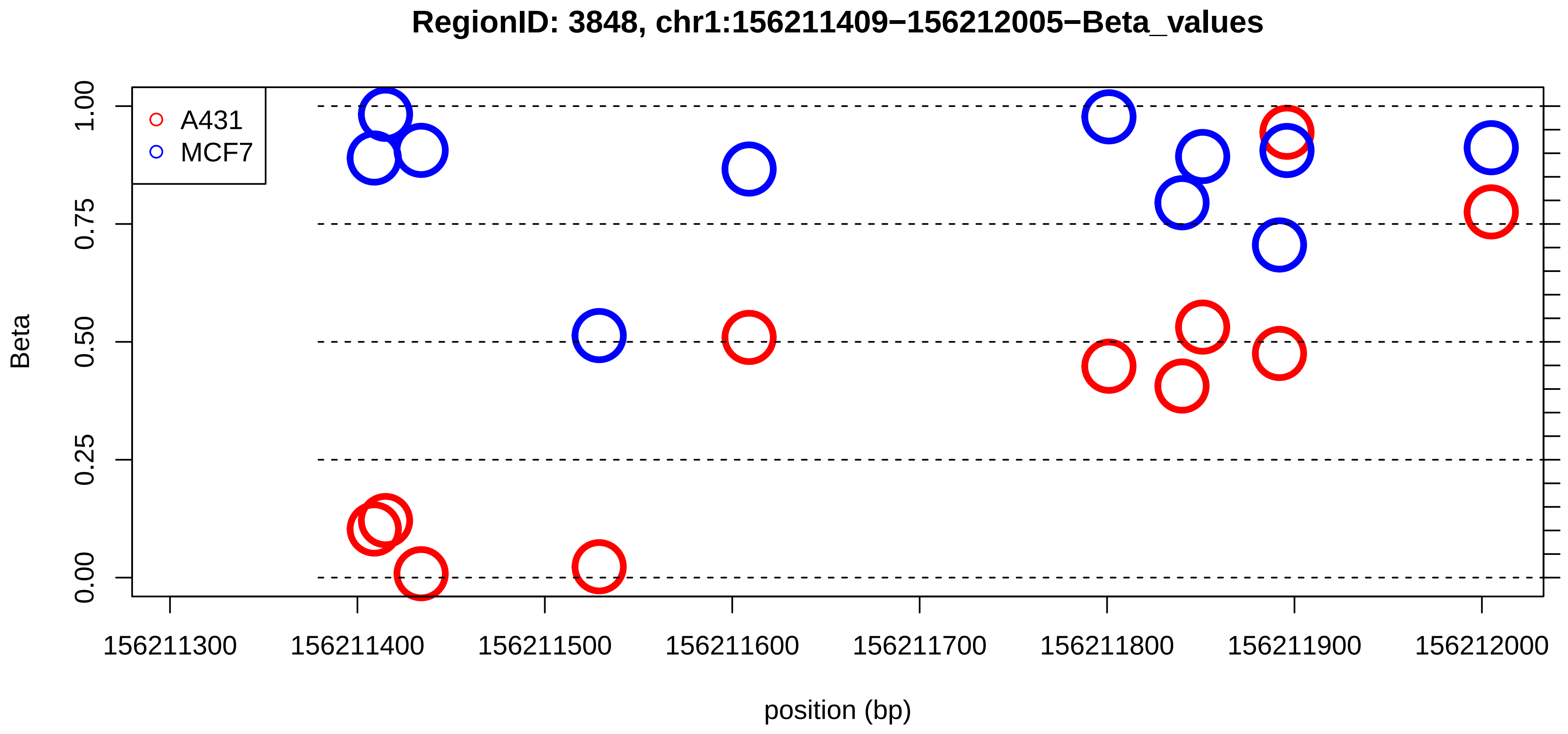
RegionID: 3848, chr1:156211409−156212005−Beta_values
A431
MCF7
Beta
1.00
0.75
0.50
0.25
0.00
156211300
156211400
156211500
156211600
156211700
156211800
156211900
156212000
position (bp)

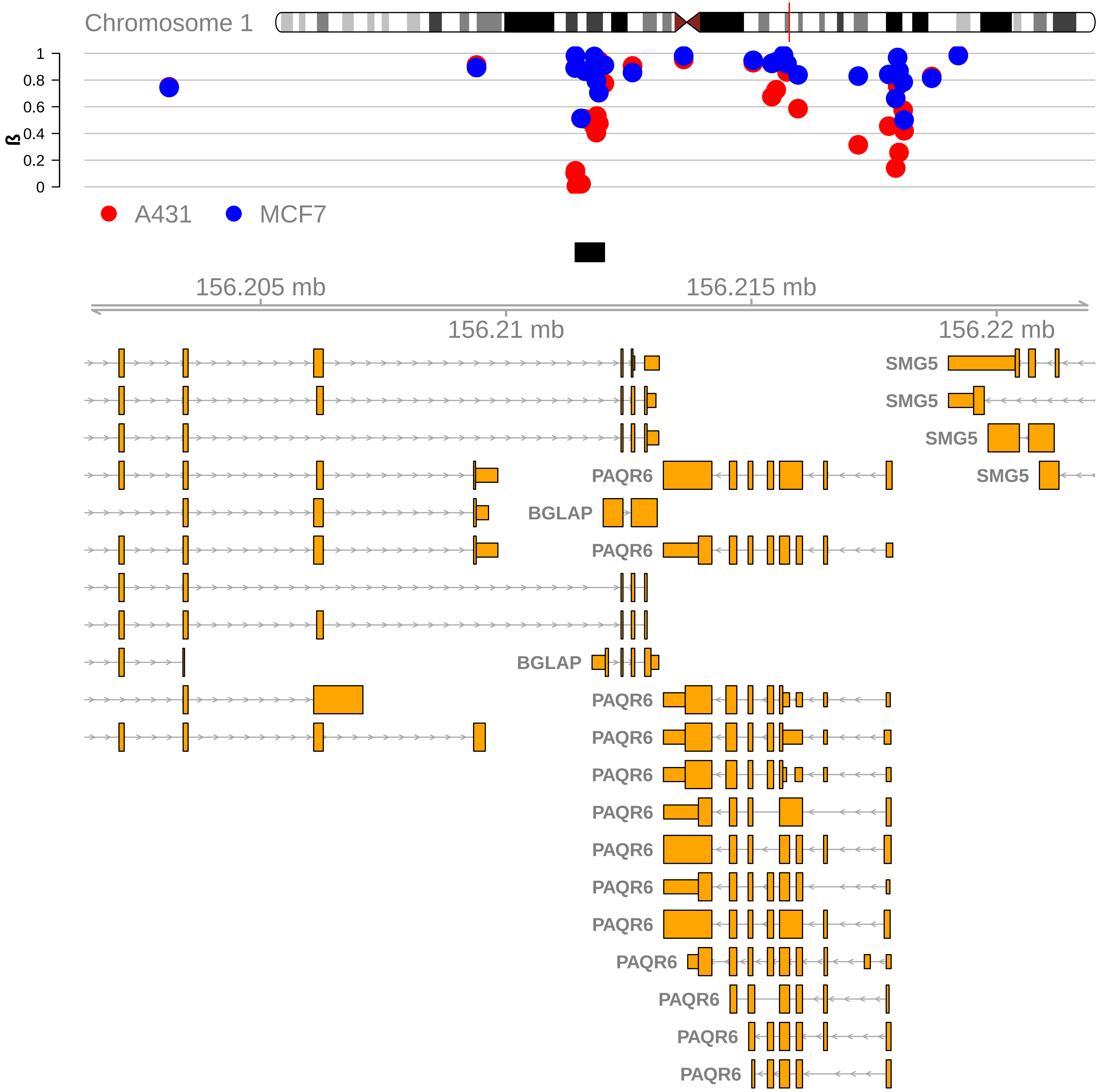
Chromosome 1
ß
1
0.8
0.6
0.4
0.2
0
A431
MCF7
156.205 mb
156.215 mb
156.21 mb
156.22 mb
SMG5
SMG5
SMG5
PAQR6
SMG5
BGLAP
PAQR6
BGLAP
PAQR6
PAQR6
PAQR6
PAQR6
PAQR6
PAQR6
PAQR6
PAQR6
PAQR6
PAQR6
PAQR6
